# The Multiplex Network of EU Lobby Organizations

**DOI:** 10.1371/journal.pone.0158062

**Published:** 2016-10-28

**Authors:** An Zeng, Stefano Battiston

**Affiliations:** Department of Banking and Finance, University of Zurich, Zurich, Switzerland; Universidad Rey Juan Carlos, SPAIN

## Abstract

The practice of lobbying in the interest of economic or social groups plays an important role in the policy making process of most economies. While no data is available at this stage to examine the success of lobbies in exerting influence on specific policy issues, we perform a first systematic multi-layer network analysis of a large lobby registry. Here we focus on the domains of finance and climate and we combine information on affiliation and client relations from the EU transparency register with information about shareholding and interlocking directorates of firms. We find that the network centrality of lobby organizations has no simple relation with their lobbying budget. Moreover, different layers of the multiplex network provide complementary information to characterize organizations’ potential influence. At the aggregate level, it appears that while the domains of finance and climate are separated on the layer of affiliation relations, they become intertwined when economic relations are considered. Because groups of interest differ not only in their budget and network centrality but also in terms of their internal cohesiveness, drawing a map of both connections across and within groups is a precondition to better understand the dynamics of influence on policy making and the forces at play.

## Introduction

Lobbies express the interest of specific groups within the society and the economy (e.g., banks or consumers) and their pressure on regulators constitute part of the process by which policies are shaped [1–3]. Indeed, the study of interest group politics has long been an important subfield of political economy. In particular, the problem of regulation is seen there as “discovering when and why an industry or other group of likeminded people is able to use the state for its purposes” [4]. On the one hand, social pressure (in particular from NGO) can be a crucial determinant to induce firms to better deal with the negative externalities of their activities. On the other hand, social pressure creates an incentive for firms to act collectively against the activists [5] (e.g. with green-washing initiatives). In this respect, it has been emphasized that firms have incentives to put in place sophisticated strategies such as artificial grassroots campaigns, in order to distort the information provided to regulators [6]. In particular, the relation between negative externalities such as pollution and market power has been analysed in the sector of non-renewable resources [7].

Many countries enforce or encourage lobby activities to be disclosed and several register exist (such as the US register at the House of Representatives, or the EU transparency register), from which reports are compiled regarding the size, the number and composition of lobbies. However, little research has been carried out so far on the structure of the networks in which lobbies operate [8]. This is somewhat surprising since we can expect the network structure of lobbies to play an important role for at least two reasons. First, lobbies can be thought of as intermediaries between economic and social groups on the one hand and policy making bodies on the other hand. Therefore, the influence they are able to exert could depend on the arrangement of ties their ties to economic and social actors, political parties, and policy making bodies. Second, a cohesive group of lobby organizations could be more effective in influencing policy making than a disperse collection of lobby organizations [9]. Given the increasing importance of empirical studies of networks [10, 11], in this paper we aim to fill the gap in the literature. While here we do not examine the success of lobbies in exerting influence on specific policy issues, we perform what to our knowledge is the first systematic characterization from an empirical perspective of a large lobby network. Such a characterization is a precondition to further studies that should be aimed at better understand the dynamics of influence on policy making and the forces at play [12]. In particular, drawing a “map” of lobbies and measuring their cohesion and fragmentation may help policy makers to better deal with the pressure from lobbies. Conversely, public interest lobbies, which are typically outnumbered by industry lobbies, can see how to get better heard in the policy making process.

In summary, we construct a multiplex lobby network [13–16] by combining the data from the EU transparency register and the Bureau van Dijk database Orbis. The multiplex network consists of four layers of different types of relations, including affiliations among organizations, client-customer relations, interlocking directorates and stock shares ownership relations. We focus mostly on the domains of climate and finance, as paradigmatic cases. We first study the structure of each network layer and we then consider the network as whole. We compute centrality measures, both on individual network layers and taking into account all layers, as proxies for the potential influence of organizations in the lobby network. We find that while the budget of an organization to represent interests (hereafter, “lobby money”) is certainly important, it is not a good predictor of the centrality of the organization in the lobby network. Because we can reasonably expect centrality to affect the ability of lobby organizations to exert influence, this result suggests that there is more to influence than simply the budget and that the position in the network structure should matter. We further proceed the analysis from a microscopic level (individual organizations) to a mesoscopic level (10 different groups, e.g. banking, insurance, energy, etc.), to a macroscopic level (finance and climate). We find that certain structural properties can only be appreciated in the multiplex network and not in the single layers. For instance, the inter-group and inter-domain interactions [17] become visible only when the shareholding and interlocking network level are taken into account. At the group level, we propose to complement the statistics on budget with those on cohesiveness, the rational being that groups that have at the same time large budget but also strong internal cohesiveness have more chances to exert influence.

## Results

We consider the 6637 lobby organizations in the EU transparency register updated at November 2014 and we construct a multiplex network (hereafter “lobby network”) among them consisting of four different layers conveying information and economic interests in a specific way as described further below. Three layers, i.e. affiliation, shareholding and client are directed networks, while the interlocking level is undirected. Shareholding and interlocking networks are in principle weighted, but for simplicity we regard them as unweighted networks in this paper. The data collection procedure is described in the Methods section.

We look at three different scales. The microscopic scale of the individual organizations is the scale at which one usually considers a network. At the macroscopic scale, we are interested in how broad policy domains are related and in this paper we focus only on finance and climate. We further look at a mesoscopic scale by categorizing organizations into groups of economic interest (e.g. banking, insurance etc. in finance; and energy, utilities, etc. in climate). In the following, we first provide some basic statistics of the lobby network at the microscopic level. We then investigate the relation between network measures and quantities related to the lobbying effort of groups and organizations (e.g. money and number of representatives). We further propose a centrality measure to study the influence of lobby organizations in the network. Finally, we analyze the connectivity patterns of the lobby network at the mesoscopic and macroscopic levels.

### Lobby Money and Network Measures

**Fig 1:**
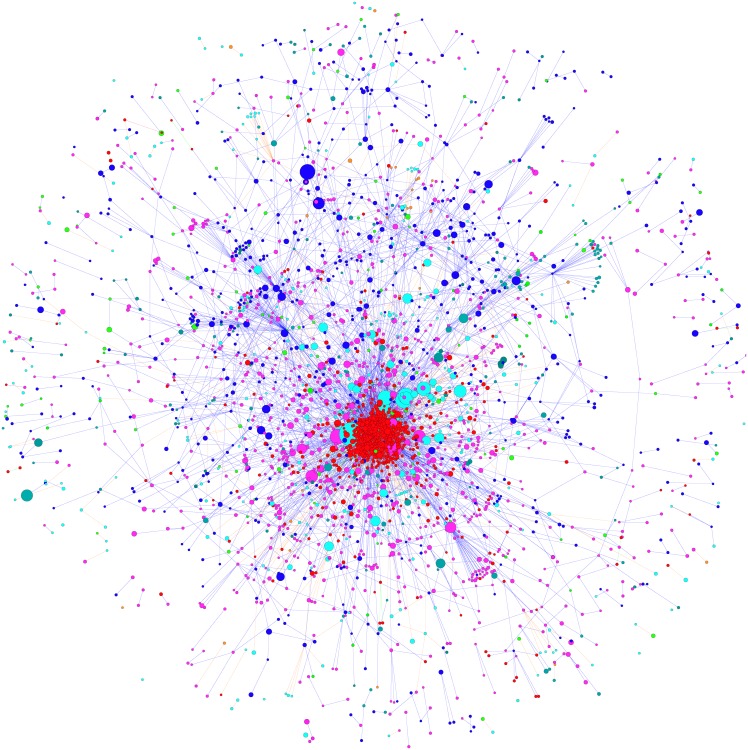
The visualization of the lobby network. The organizations of different types are marked in different colors as follows, red: companies; pink: trade, bussiness & professional associations; cyan: professional consultants and law firms; blue: NGOs; green: think tanks and research institutions. The size of the node is proportional to the number of lobbyists of the organization.

Organizations are registered in one of the following 13 categories: companies; trade, business&professional associations; trade unions; other similar organisations; professional consultancies; law firms; self-employed consultants; non-governmental organisations, platforms and networks and similar; think tanks and research institutions; academic institutions; organisations representing churches and religious communities; local, regional and municipal authorities; other public or mixed entities. The visualization of the lobby network is shown in Fig 1. Each category is marked by a different color. In the network, the companies (in red) appear to be tightly connected by the shareholding and interlocking links. Both companies and NGOs (in blue) are affiliated to some business associations and trade unions (in pink). They are also clients of the professional consultants and law firms (in cyan). Qualitatively, the companies appear to form a core in the middle of the network while the NGO’s are scattered in different parts of the network. The detailed structure of the shareholding and interlocking network layers is shown in Fig A in S1 File. One can see that companies tend to be clustered by countries in line with previous work on ownership and geography [18].

The basic statistical properties of each layer of the lobby network are shown in Table 1. The affiliation layer has the largest number (i.e. 2472) of nonisolated nodes while the shareholding layer has the largest number (i.e. 4175) of links. The degree distribution in the shareholding and affiliation networks is very heterogeneous while the degree distribution in the interlocking and client networks is much less heterogenous (see Fig B in S1 File). This is also shown by the the reciprocal of the Herfindahl index of the degree sequence (*H* = (∑*k*)^2^/∑(*k*^2^)) in Table 1 which measures the number of leading nodes (with respect to degree) in the networks. In the shareholding and affiliation networks, the fraction of significant nodes (i.e. *H*/*N*) is low, indicating that a small number of nodes attract most of the links. In the interlocking and client networks, the fraction of significant nodes is higher, indicating that most of the nodes have similar degree. Due to the high link density in the shareholding network, the average shortest path is low (2.12) and clustering coefficient is high (0.63) in this network layer. In the affiliation, shareholding and client layers, the degree assortativity is negative (−0.16, −0.61,−0.26 respectively), indicating that small degree nodes tend to connect to high degree nodes. On the contrary, the degree assortativity in the interlocking layer is positive (0.32). This means that big organizations tend to have common directors with each other rather than with small organizations [19]. The relations between node degree in different layers are shown in Fig C in S1 File.

**Table 1:** The basic statistical properties of each layer of the lobby network. *N* is the number of nonisolated nodes. *E* is the number of links. 〈*k*〉 is the average degree of the network. *H* is the reciprocal of the Herfindahl index of the degree sequence. *d* is the average shortest path length of the network. *c* is the average clustering coefficient of the network. *r* is the assortativity coefficient of the network. We consider these networks as undirected when calculating these indices.

layer	*N*	*E*	〈*k*〉	*H*	*d*	*c*	*r*
affiliation	2472	3221	1.3	210	6.57	0.04	-0.16
shareholding	383	4175	10.9	23	2.12	0.63	-0.61
interlocking	378	862	2.28	209	4.37	0.17	0.32
client	573	467	0.8	110	6.82	0	-0.26

**Fig 2:**
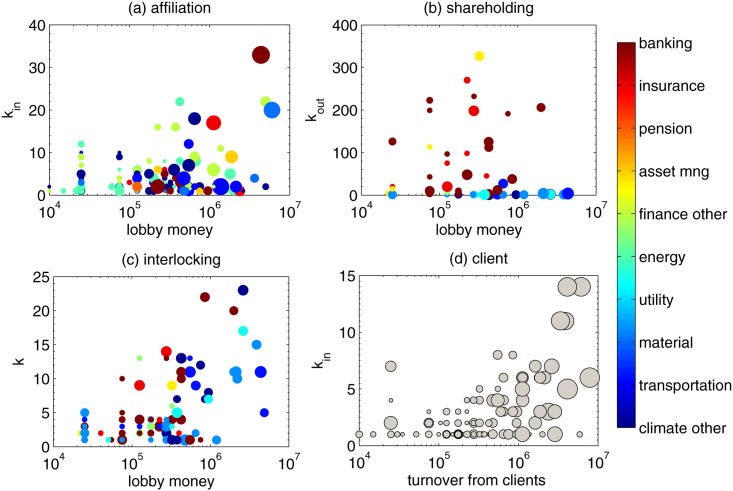
(a) Scatter plot of nodes’ in-degree in the affiliation network (i.e. number of members) versus the lobby money (i.e. estimated cost). (b) Scatter plot of nodes’ out-degree in the shareholding network (i.e. number of held companies) versus the lobby money. (c) Scatter plot of nodes’ degree in the interlocking network (i.e. number of organizations with at least one common management) versus the lobby money. (d) Scatter plot of nodes’ degree in the client network (i.e. number of clients) versus the turnover representing the interests of clients. The size of each bubble is proportional to the number of lobbyists of the organization. Organizations in different groups are marked by different colors. The organizations in (d) are not colored because they are law firms and professional consultants, and not assigned to any of the group.

We then move to study the relation between “lobby money” and other properties of the lobby organizations. Hereafter, lobby money refers to the “estimated costs” of each organisation “directly related to representing interests to EU institutions in the last year”. Notice that this number is an estimation provided by the organizations themselves. The relations between lobby money and organizations’ operating revenue and total assets are shown in Fig D in S1 File. In Fig 2 we also study the relation between the connectivity degree of organizations on the y-axis and their “lobby money” on the x-axis in different network layers. In the figure, the bubble size represents the number of lobbyists in each organization.

In general, we do not observe clear patterns in the relation between degree and lobby money. Although, in some cases the Pearson correlation turns out to be around 0.5, the scatter plots show that organizations with the same degree can have lobby money varying across several orders of magnitude. In the affiliation layer, the nodes with larger out-degree (i.e. number of members) show some tendency to have larger lobby money. However, organizations with similar out-degree can have very large variance in the lobby money and the Pearson correlation is rather low, i.e. 0.46. The situation is similar in the interlocking layer, with the Pearson correlation between the nodes’ interlocking degree and lobby money equal to 0.52. In the shareholding network, the correlation between the out-degree (i.e. number of held companies) and lobby money is very weak, with a Pearson correlation of −0.15. Notice that the large out-degree organizations are all finance organizations. Finally, in the client layer, the lobby money is not available and law firms and professional consultants report instead the “turnover realized by the organization in representing the interests of clients”. The Pearson correlation of in-degree (number of clients) vs turnover is 0.67. The organizations with large in-degree have some tendency to have both large turnover and number of lobbyists.

Overall, the results indicate that lobby money is not a good predictor of the connectivity degree of organizations in the lobby network. At a first thought, this could be surprising since one could expect the organizations with larger budget to be also more connected in the various networks. To further investigate this point, we analyze the centrality of the organizations.

### Centrality

We want to capture the influence of each organization in the lobby network by means of a network centrality measure. Following a common approach in the literature we use a feedback-centrality akin to the Katz centrality [20] and to Page-Rank [21]. In particular, there are two ways to investigate the centrality of nodes. For instance, in the affiliation network, one can consider the in-centrality in which an organization is important if, recursively, it has many important members. We can also consider the out-centrality in which an organization is important if, recursively, it is affiliated to and thus influencing many important organizations. Similarly, in the ownership network layer, the out-centrality measures the importance of shareholders, while the in-centrality of a company is higher if it has many important shareholders. The notion of centrality can be then extended to the multiplex network by including all the link types: affiliation, shareholding and interlocking relations [13–16, 22, 23] (see Methods). In this case, the centrality is based on a iterative process across all layers in the network. By accounting for this layer-layer interaction, a less connected node in one layer might eventually turn out to have high centrality if it is well connected in other layers. Therefore, the latter way of computing centrality captures the interaction between the layers in the multiplex network.

**Fig 3:**
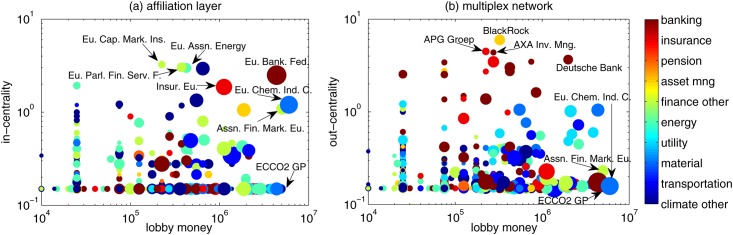
(a) Scatter plot of node’ in-centrality in the affiliation network versus the lobby money. (b) Scatter plot of node’ out-centrality in the multiplex lobby network versus the lobby money. The size of each bubble is proportional to the number of lobbyists of the organization. Organizations in different groups are marked by different colors.

The scatter plot of organizations’ in-centrality in the affiliation network versus their lobby money is shown in Fig 3(a) and the scatter plot of organizations’ out-centrality in the multiplex network versus their lobby money is shown in Fig 3(b). In general, there is no simple relation between centrality and lobby money (the Pearson correlation coefficients in these two figures are respectively 0.172 and 0.074). This means that lobby money is not a good predictor of how influential organizations are (assuming of course that the centrality as defined here captures to some extend the influence of organizations). This also indicates that information on centrality of organization is a valuable quantity that complements the information on their lobby money. Therefore, neglecting the network structure (as in most of the literature) will miss a large amount of information when analyzing the influence of lobby organizations.

Overall, the scatter plots in Fig 3 provide the following information. While large lobby money does not imply large influence, there are some organizations that have both large lobby money and centrality, such as the European banking federation in Fig 3(a) and the Deutsche Bank in Fig 3(b). This could be interpreted as an indication that they both have the financial means and the social capital to influence the policy making process around topics in which they have an interest. The names of few top players are displayed in Fig 3. The names of the top 20 players are listed in Table B in S1 File.

Interestingly, the organizations with high in-centrality are not the same as those with high out-centrality. For the in-centrality, the top players are mainly professional associations involved, respectively, in energy, financial markets, insurance and banking. This reflects of course the definition of in-centrality because such professional organizations have memberships from many other important organizations. For the out-centrality, the top players are mainly companies in either asset management, or banking. This is due to the fact that they hold shares of many important companies.

### Connectivity patterns at meso- and macro-scale

**Fig 4:**
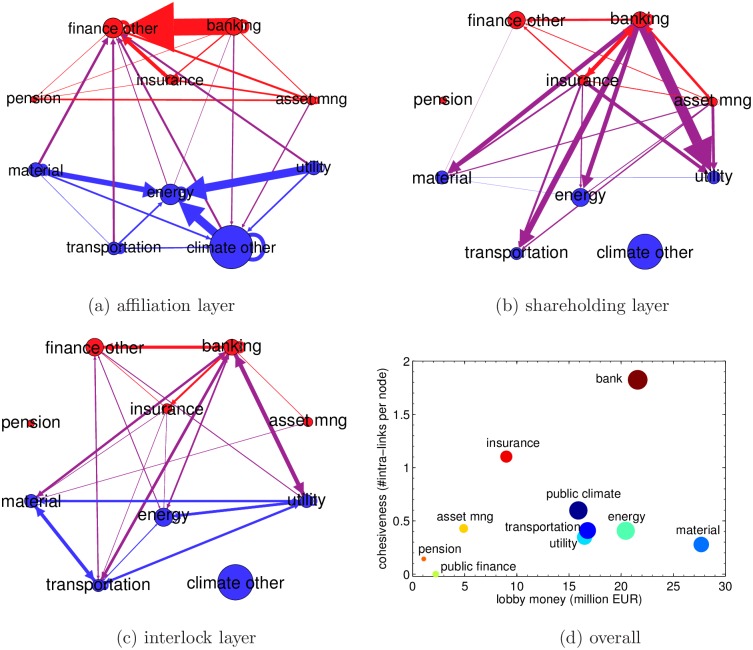
(a) the affiliation links between different groups. (b) the shareholding links between different groups. (c) the interlocking links between different groups. (d) The scatter plot of the lobby cohesiveness versus the lobby money within groups. The size of the bubbles in (a)(b)(c) is proportional to the number of organizations in the group. The size of the link between two groups is proportional to the number of links from the organizations in one group to the organizations in the other group in the original network. The size of the points in (d) is proportional to the total number of lobbyists of the group.

We further investigate the lobby network at the mesoscopic and macroscopic levels [24], see results in Fig 4. As mentioned above, at the macroscopic level, the organizations are selected if they belong to two paradigmatic domains on which we focus in this paper, namely the finance domain and climate domain. By climate domain, we actually mean CO2-intensive sectors, but we use the climate label for brevity. Accordingly, at the mesoscopic level the organizations are classified into 10 groups of economic interest related to the financial sector (i.e. “banking”, “insurance”, “pension”, “asset management”, “finance other”) and climate sectors (i.e., “energy”, “utility”, “material”, “transportation”, “climate other”), as shown in Table 2. If they do not belong to any of these groups, they are discarded from the analysis. In this section, we study the connectivity pattern in the group networks and we discuss the relations within and across the two domains.

**Table 2:** The number of links within each group in the empirical networks (i.e. *links*) and the randomly reshuffled networks (i.e. links*).

		affiliation		shareholding		interlock		overall	
group	size	*links*	links*	*links*	links*	*links*	links*	*links*	links*
banking	75	43	10.3±2.8	84	84.3±3.6	4	5.1±1.6	131	99.6±4.3
insurance	29	17	1.8±1.3	13	12.4±1.9	1	0.2±0.5	31	14.5±2.2
pension	7	1	0.0±0.1	0	0±0	0	0±0	1	0.0±0.1
asset mng	21	6	0.4±0.6	3	4.0±1.0	0	0.0±0.14	9	4.4±1.4
finance other	75	31	9.1±2.8	0	0.1±0.3	0	0.5±0.65	31	9.7±2.9
energy	83	31	7.1±1.9	0	0.1±0.3	1	0.6±0.67	32	7.8±1.9
utility	52	2	0.1±0.2	2	1.0±1.0	7	4.1±1.8	11	5.1±2.1
material	54	12	1.4±1.3	1	1.2±1.1	1	1.0±0.8	14	3.6±2.0
transportation	45	15	1.0±1.0	2	0.2±0.4	1	0.6±0.7	18	1.8±1.3
climate other	208	91	31.8±3.6	0	0±0	0	0±0	91	31.8±3.6

#### Affiliation Layer

The first network layer is the affiliation network. From the EU transparency register data we extract the affiliation relations between organizations, whereby a link from organization *i* to organization *j* means that organization *i* is a member of organization *j* (or affiliated to *j*). This type of relation reflects common economic sector activity and/or common economic interests with respect to regulation. The visualization of this network at the mesoscopic level is shown in Fig 4(a).

At the macroscopic scale, this layer displays a strong community structure [25], in the sense that links among organizations within finance and within climate are much more numerous than the links among organizations across domains. As shown in Table 3, the number of links within the finance domain is 178 and the number of links within the climate domain is 233. In contrast, the total number of links between finance and climate is only 16. Besides the number of links, we also measure a quantity called intra-link ratio which is defined as the number of links within a domain divided by the total number of links of the domain (see results in Table A in S1 File). The intra-link ratio for the finance domain is 92% and the intra-link ratio for the climate domain is 94%. In order to investigate how significant this observation is, we compare the observed results to their expected values obtained from a sample of networks where the links among the organizations are reshuffled keeping the degree sequence unchanged [26]. After the link reshuffling, the number of links within the finance and climate domain decreases from 178 to 80.8±5.8 and from 233 to 135.8±5.8 respectively, while the total number of links across domains increases from 16 to 210.4 ± 5.8. Equivalently, the intra-link ratio decreases from 92% to 28% ± 3% in finance domain and from 94% to 39% ± 2% in climate domain.

**Table 3:** The number of links within and between domains in the empirical networks (i.e. *links*) and the randomly reshuffled networks (i.e. links*). In the affiliation network, *i* → *j* means *i* is affiliated to *j*. In the sharesholding network, *i* → *j* means *i* is holding share of *j*. The interlock network is an undirected network, *i* → *j* means the number of common managers between *i* and *j*.

	within finance		within climate		finance→climate		climate→finance	
	*links*	links*	*links*	links*	*links*	links*	*links*	links*
affiliation	178	80.8±5.77	233	135.8±5.77	7	104.2±5.77	9	106.2±5.77
shareholding	281	279.4±1.77	14	12.4±1.77	400	401.6±1.77	7	8.6±1.77
interlock	19	14.0±2.1	32	27.0±2.1	31	41.1±4.2	31	41.1±4.2
overall	478	374.2±6.1	279	175.2±6.1	438	546.8±7.0	47	155.8±7.0

At the mesoscopic scale, a similar result holds. For instance, the number of links within each group both in finance and climate is much larger than what could be expected by chance. Table 1 provides the number of links in each group, as well as the average number (plus or minus one standard deviation) in the reshuffled sample. For example, the number of links within the banking group decreases from 43 to 10.3 ± 2.8 after link reshuffling (Equivalently, the intra-link ratio decreases from 43% to 8% ± 2%). Similar phenomenon is observed in other groups, see Tables 2 and 3 and Table A in S1 File. These results indicate that there is a hierarchical community structure [25] in the affiliation network, i.e. the partition based on the sector corresponds to a possible community structure both at the macroscopic and mesoscopic level.

#### Shareholding and Interlocking Layers

The second and third network layers are the shareholding network and the interlock network. We match the organizations in the EU transparency register (the subset of the companies) with the ORBIS database and we extract the shareholding and interlocking relations among them. In a shareholding relation a link from organization *i* to organization *j* means that company *i* own shares of company *j*. This type of relation reflect an interest of company *i* in the economic performance of company *j* [10]. Conversely, in the interlocking relation, a link from organization *i* to organization *j* means that company *i* and *j* share at least one director or manager in their boards. This type of relation allows companies to share information and insights on economic trends. The visualization of the shareholding and interlocking networks at the mesoscopic level are shown in Fig 4(b) and 4(c), respectively.

The interlocking network layer displays some level of community structure, although not as marked as the affiliation layer. Indeed the number of links between finance and climate are smaller than what would be expected from a random process of link formation given the degree sequence. Yet the number of links is still comparable to the number of links within finance and within climate. After link reshuffling, the number of links within finance and within climate decreases from 19 to 14 ± 2.1 and from 32 to 27 ± 2.1, respectively (the intra-link ratio thus decreases from 34% to 24% ± 4% within finance and from 52% to 43% ± 3% within climate). The links between finance and climate, on the contrary, increases from 31 to 41.1 ± 4.2. Thus, at the level of groups, the interlocking links are very sparse within each group and the evidence of community is less obvious.

Note that that there is an entire stream of works on both interlock and ownership networks and that our study here only focuses on a subset of the interlock and ownership network, namely the one consisting of links among firms that at the same time have their own representative advocacy office appearing in the EU lobby register. Our results are in line with those found in the literature. While earlier studies reported on statistical properties of these networks in selected countries [27–30], more recent works analyzed the conditional deviations from randomness of the interlock network [31], and the resilience of its core with respect to policy reforms [32–34].

In contrast with the interlock and with the affiliation layer, the shareholding layer displays many inter-domain linkages. In particular, the number of relations from finance to climate is even larger than the number of relations within the finance domain. As shown in Table 3, the number of links from finance to climate is 400 while the number of links within finance is 281. The relations from climate to finance are instead very few. There are only 7 such links. This situation reflects the tendency of financial companies to invest in real sector companies. Similar results hold for economic groups: for instance the number of links from banks to other groups in the climate domain are more numerous than those within the banking group. Specifically, there are 230 links from banks to groups in climate and 84 links from banks to banks. Similarly, insurance and asset management hold many more shares in groups such as utilities and materials than in their own groups. However, they hold many shares in the banking group. For the insurance group, the number of links to climate organizations, banks and itself are respectively 100, 42 and 13. For the asset management group, the number of links to climate organizations, banks and itself are respectively 69, 29 and 3. Interestingly, both the results at the domain and at the group level are consistent with what would be expected from the network reshuffling. This means that the degree sequence drives most of the structure in this network layer.

#### Client Layer

The fourth network layer is the client network. From the EU transparency register we also extract the client relations, where a link from organization *i* to organization *j* means that organization *i* is a client of organization *j*, which can be either a law firms or a professional consultant. This type of relation reflects the fact that the law firm/ consultant is acting in the interest of the client organization with respect to the European Commission. The visualization of this network at the mesoscopic level is shown in Fig E in S1 File.

Law firms and professional consultants (LFPC) acting in the interest of organizations in the finance and climate domain are a group of 72 organizations. The network structure is simply a star network in which all groups have linkages pointing to LFPC in the center. The four groups with highest number of links to LFPC are climate other (34 links), finance other (18 links), banking (14 links) and material (14 links). In order to remove the size effect, we also calculate the mean degree of each group, the four groups with the highest client degree are insurance (〈*k*〉 = 0.28), material (〈*k*〉 = 0.26), finance other (〈*k*〉 = 0.24) and banking (〈*k*〉 = 0.19).

#### Lobby cohesiveness

The advantage of considering the system as a multilayer network is that one can understand the relation between lobby organizations in a more comprehensive way. The single layer case can only provide one type of relation between lobby organizations, while the multilayer network can show different types of interactions between organizations. For instance, with only the shareholding layer, one can only see that the banking sector is holding shares of other sectors. What one would miss out in this case is that in the affiliation layer sectors have very strong community structure such that the links in reality are not only pointing from banks to other lobby organizations. With a complete picture of the network, one can understand better which sector has strongest lobby power.

We finally aggregate links of different types and calculate the total number of links within each groups (i.e. intra-links). In the “climate other” and in the “finance other” groups, we extract manually the organizations representing the public interest, based on their profile and declared goals in the register, and we denote them respectively as “public climate” group (87 organizations) and “public finance” group (6 organizations). We then compute the number of intra-links per node for each group and we use it as a measurement for the group’s lobby cohesiveness. In Fig 4(d), we present the scatter plot of the lobby cohesiveness versus the lobby money of the group. We see that the lobby cohesiveness of most of the groups are around or below 0.5 except banking and insurance groups. The public climate and public finance group both have low lobby money and lobby cohesiveness. Even though banking and insurance groups do not have the highest lobby money, their lobby cohesiveness is the highest (1.8 and 1.1 respectively).

## Concluding remarks

In this paper, we combine the data from the EU transparency register and the Orbis database to construct a multiplex lobby network consisting of the affiliation, shareholding, interlocking and client relations between lobby organizations. We first study the lobby network at the microscopic level. The degree distribution in the shareholding and affiliation networks is very heterogeneous while the degree distribution in the interlocking and client networks is much less heterogenous. The most densely connected layer is the shareholding network layer. The affiliation, shareholding and client networks show certain level of disassortativity while the interlocking network shows certain level of assortativity. However, no clear pattern is observed in the relation between degree and lobby money. We also propose a centrality measure to capture the influence of organizations in the lobby network. There are different notions of influence which can be captured by in-centrality and out-centrality. In both cases, we find that lobby money is not a good predictor of the centrality. While large lobby money does not imply large influence, there are still some important organizations that have both large lobby money and centrality. Moreover, we find that out-centrality is not a good predictor of in-centrality. In particular, the organizations with high in-centrality are mainly professional associations while the organizations with high out-centrality are mainly banks and asset management companies.

We finally investigate the lobby network at the mesoscopic and macroscopic levels. At the mesoscopic level, the organizations are classified in 10 different groups (e.g. banking, insurance, energy, etc.) according to the economic interest. The organizations are further classified in either the finance domain or climate domain at the macroscopic level. By studying the connectivity pattern between different groups and the relations within and across domains, we find that adding the shareholding and interlocking links from the online Orbis database is very important for understanding the interactions between lobby organizations. At both mesoscopic and macroscopic levels, we find that there is an obvious community structure in the affiliation network, i.e. the interactions between different groups and especially between different domains are very few. However, cross-group and cross-domain interactions are instead quite strong shareholding and interlocking links are considered. We observe that shareholding links are mainly from banks towards companies in the climate domain. In addition, differently from the other three networks, the shareholding network has a special feature that it is very robust to the link reshuffling at the mesoscopic and macroscopic levels. After taking into account the shareholding and interlocking links, the banking group and insurance group turn out to have highest intra-group mean degree, indicating that these two groups are the most cohesive ones in the lobby network.

In general, finding an appropriate description of the interaction between layers in a multiplex network and show if and when does it make a difference to account for the interaction are central challenges in the field of complex networks. On the one hand, the paper investigates each layer separately and compute centrality based on the links in one layer. On the other hand, we also compute the centrality of lobby organizations accounting for all the layers together. Accounting for this layer-layer interaction, lead to two types of insights that could not be obtained from the single layer analysis alone. First, a less connected node in one layer might eventually turn out to have high centrality if it is well connected in other layers. Second, there are group of nodes or sectors that appear to be interconnected only once several types of links are considered. For instance, based on affiliation links, the climate and finance arenas appear disconnected. However, when shareholding and interlock are also considered they appear very much entangled, which is crucial for policy implications. Of course, several other layers exist which are not captured in the present study. However, our results suggest that neglecting the multiplex nature of the lobby network would lead to misleading conclusions on the relations among sectors.

The analysis carried out in this paper is based on the available snapshot of the multi-layer network between lobby organizations. One interesting extension is to investigate how the evolution of a given layer influences that of other layers, which asks for further data collection and analysis in the future.

## Methods

**Data collection.** The “EU transparency register” (http://ec.europa.eu/transparencyregister/) is publicly available in xml format. We extracted the basic information of all the 6637 organizations that have registered. Then we extracted two types of links between these organizations as follows.

- **Affiliation layer.** In the EU transparency register data (xml file), each organization reports its member organizations in the “structure” section. For each organization *i*, we extract the names from the “structure” section and then do the exact matching with all the names and acronyms of the 6637 organizations. Once there is an exact string matching, we create an affiliation link from the matched organization to *i*, meaning that the matched organization is affiliated to the target organization. Besides, each organization reports in the “networking” section the organizations that it belongs to. The information in the“networking” part is composed of free text. For each organization *i*, we do a string match of all the 6637 organization names in the free text. If any organization name is matched exactly, then an affiliation link is created from *i* to the matched organization.
- **Client layer.** In the xml file, the law firms and professional consultant companies list some of their clients. For each law firm or professional consultant company *i*, we do the exact string matching between the listed client names and all the 6637 organization names. If a match is found, we construct a client link from the matched organization to *i*.

We then combined the information in the EU transparency register with the online ORBIS database available under commercial license from Bureau van Dijk. We selected all the organizations registered as companies. The company names were uploaded to the online ORBIS database. When several possible matchings were found, the selection was done manually, based on the profile of the company in the registry and its financial data such as operating revenue. For the matched companies, we extract some basic financial information, including the operating revenue, total assets, board directors, and shareholders.

- **Interlocking layer.** The interlocking links are constructed based on the common managers between each pair of companies. Therefore, the interlocking network is weighted, with the link weight equal to the number of common board directors.
- **Shareholding layer.** The shareholding links are the shareholding relations between the above matched companies. This network is again a directed weighted network. The weight of the link is the percentage of shares that a company is holding in the other company.

Note that both data sources do not provide historical information, so it is only possible to study one time snapshot at this stage.

## Centrality measure

We design a simple feedback-centrality measure [21] to try and quantify the influence of each organization in the lobby network. The in-centrality of a node is defined as

$$c_{i}=\left(1-\alpha \right)+\alpha \sum_{j}w_{ji}\left(c_{j}+m_{j}\right),$$ (1)

In the definition above *m*_*j*_ represents the intrinsic importance of the nodes and it is proxied by the relative lobby money of node *j*, i.e. *m*_*j*_ = *s*_*j*_/∑_*h*_(*s*_*h*_), where *s*_*h*_ is the original lobby money of node *j*. The matrix *w*_*ji*_ is a stochastic matrix, with $w_{ji}=\frac{a_{ji}}{\sum_{h}a_{jh}}$, where *a*_*ji*_ is a component of the network adjacency matrix (*a*_*ji*_ = 1 means that there is a direct link from *j* to *i*, and 0 otherwise). *α* is a constant (*α* = 0.85) similar to the dumping factor in the PageRank algorithm [21]. The influence of block *i* can be measured by *c*_*i*_ after it reaches the stationary value. According to the definition, the centrality of a node is co-determined by the centrality and lobby money of the neighboring nodes, but independent of its own lobby money.

If *w*_*ji*_ is changed to *w*_*ij*_ in Eq 1, the obtained *c*_*i*_ measures the out-centrality of nodes *i*. The current centrality definition can be easily extended to the multiplex lobby network consisting of more than one type of links [16]. In this case, *w*_*ji*_ should be computed as $w_{ji}=\frac{a_{ji}^{a}+a_{ji}^{s}+a_{ji}^{i}}{\sum_{h}\left(a_{ji}^{a}+a_{ji}^{s}+a_{ji}^{i}\right)}$ where $a_{ji}^{a}$, $a_{ji}^{s}$ and $a_{ji}^{i}$ represent the adjacency matrix for the affiliation layer, shareholding layer and interlocking layer, respectively.

## Supporting Information

S1 File Supplementary Information of “The multiplex network of EU lobby organizations”. (PDF)
