# Supplementary material for: The Multiplex Network of EU Lobby Organizations
Source: PLoS One. 2016 Oct 28;11(10):e0158062. doi: 10.1371/journal.pone.0158062 (PMC5085054; doi:10.1371/journal.pone.0158062)
Supplement: S1 File — (PDF) [file pone.0158062.s001.pdf]

Supplementary Information of "The multiplex network of EU lobby organizations"

An Zeng and Stefano Battiston

I. TABLES

Table A, the intra-link ratio within each group in the empirical networks (i.e. f) and the randomly reshuffled networks (i.e. f^*). The intra-link ratio is defined as the number of links within a group divided by the total number of links connect to the group.

		affiliation		shareholding		interlock		overall	
group	size	f	f^*	f	f^*	f	f^*	f	f^*
banking	75	43%	8%±2%	18%	19%±1%	11%	14%±5%	22%	16%±1%
insurance	29	45%	4%±3%	6%	6%±1%	10%	2%±5%	12%	5%±1%
pension	7	11%	0%±2%	0%	0%±0%	0%	0%±0%	11%	0%±2%
asset mng	21	26%	2%±2%	2%	3%±1%	0%	2%±14%	5%	3%±1%
finance other	75	30%	7%±2%	0%	0%±1%	0%	4%±5%	18%	5%±2%
finance domain	207	92%	28%±3%	41%	41%±0%	38%	26%±5%	51%	36%±1%
energy	83	33%	6%±2%	0%	0%±0%	7%	5%±5%	17%	4%±1%
utility	52	7%	0%±1%	1%	1%±1%	23%	12%±6%	5%	2%±1%
material	54	31%	3%±3%	1%	1%±1%	5%	6%±5%	9%	2%±1%
transportation	45	56%	3%±3%	2%	0%±0%	5%	3%±3%	13%	1%±1%
climate other	208	64%	16%±2%	0%	0%±0%	0%	0%±0%	64%	16%±2%
climate domain	442	94%	39%±2%	3%	3%±0%	51%	40%±4%	38%	21%±1%

Table B, the top 20
organizations of the rankings based on lobby money, in-centrality in the affiliation network and out-centrality in the multiplex network, respectively.

rank	Lobby money (estimated cost)	In-centrality (affiliation network)	Out-centrality (multiplex network)
1	European Chemical Industry Council	European Capital Markets Institute	BlackRock
2	Association for Financial Markets in Europe	European Parliamentary Financial Services Forum	APG Groep N.V.
3	ECCO2 Global Partners	Association Europenne de lEnergie	AXA Investment Managers
4	ExxonMobil Petroleum & Chemical	European Environmental Bureau	Deutsche Bank AG
5	European Banking Federation	Green 10	BPCE
6	Shell Companies	European Banking Federation	Allianz SE
7	GDF SUEZ	European Forum for Renewable Energy Sources	BNP Paribas Cardif
8	Verband der Chemischen Industrie	Insurance Europe	Socit Gnrale
9	Bundesverband der Energie- und Wasserwirtschaft	Friends of the Earth Europe	Henderson Global Investors
10	EUROGAS aisbl	European Financial Reporting Advisory Group	UniCredit
11	TOTAL S.A.	Deep Sea Conservation Coalition	Prudential plc
12	Mediterranean Information Office for Environment...	European Chemical Industry Council	Bank of America
13	Daimler Aktiengesellschaft	Forum for the Automobile and Society	The Bank of New York Mellon
14	Gesamtverband der Deutschen Versicherungswirtschaft	European Environmental Citizens Org...	Commerzbank AG
15	E.ON SE	Climate Action Network Europe	Nordea Bank AB (publ)
16	Bundesverband deutscher Banken	Association for Financial Markets in Europe	Standard Life plc
17	European Automobile Manufacturers Association	European Fund and Asset Management Association	Intesa Sanpaolo
18	ELECTRICITE DE FRANCE	European Water Partnership	Danske Bank
19	RWE AG	Council of European Energy Regulators	BKK AS
20	Deutsche Bank AG	Ind. Council research on Packaging & Environment	Agder Energi

II. FIGURES

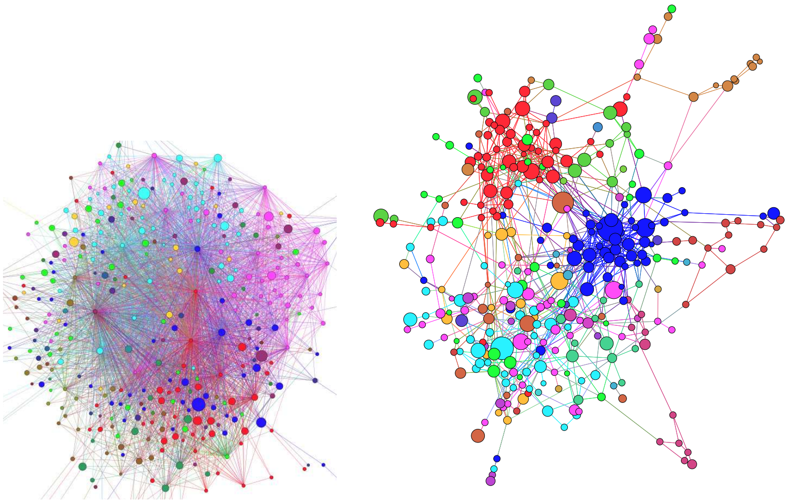

Figure A. The visualization of the (a) shareholding network and (b) interlocking network. The organizations in one country is given the same color as follows, red: France; blue: Germany; cyan: US; pink: UK; green: Belgium; orange: Switzerland. The size of the nodes is proportional to the number of lobbyists of the organizations.

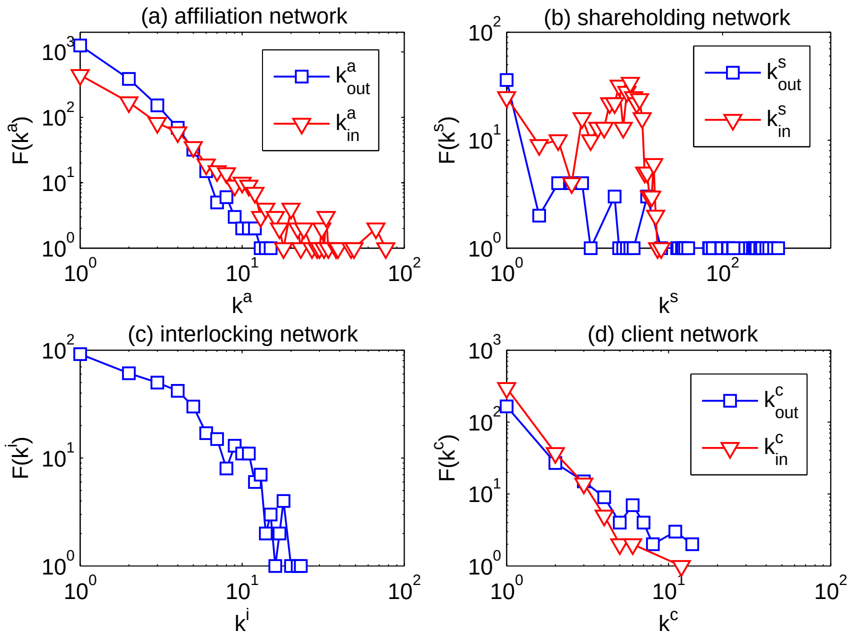

Figure B. The degree distribution of different network layers. $F(k)$ is the number of nodes with degree k.

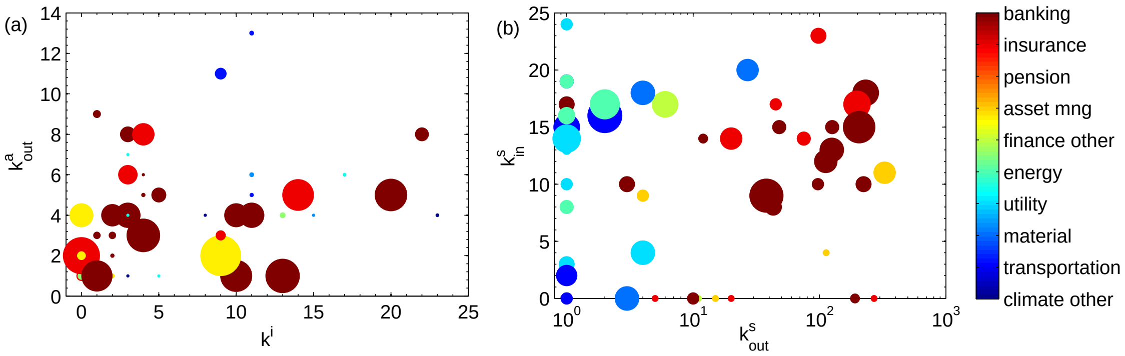

Figure C. (a) Scatter plot of the affiliation out-degree versus the interlocking degree. The size of the bubble is proportional to the shareholding out-degree of the organization. (b) Scatter plot of the shareholding in-degree versus the shareholding out-degree. The size of the bubble is proportional to the interlocking degree. k^i represents the interlocking degree, k^a_{out} represents the out-degree in the affiliation network, k^s_{in} and k^s_{out} represent the in-degree and out-degree in the shareholding network, respectively.

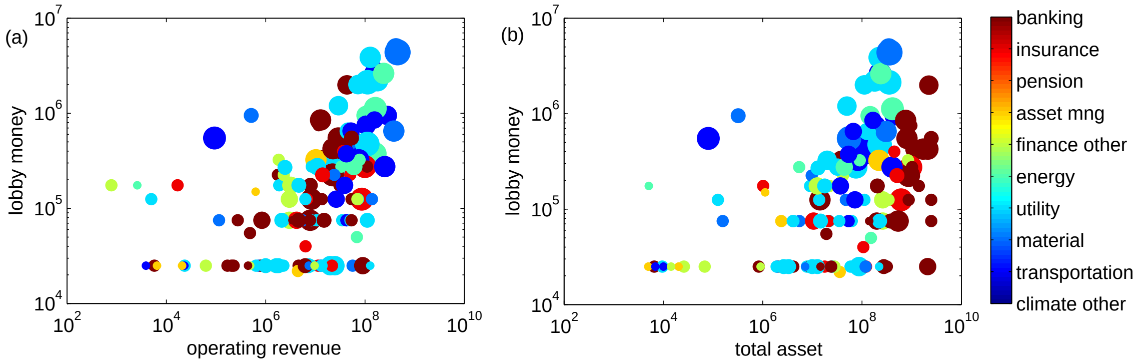

Figure D. (a) Scatter plot of companies' lobby money versus their operating revenue. (b) Scatter plot of companies' lobby money versus their total asset. The size of the bubble is proportional to the number of lobbyists of the organization.

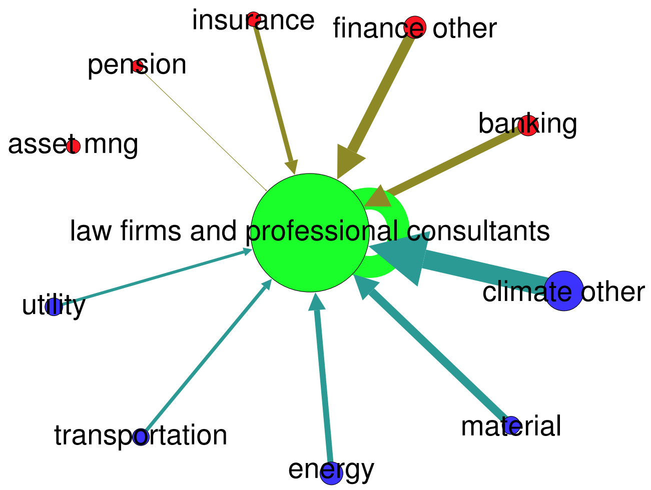

Figure E. The client links between different groups. The size of the node is proportional to the number of organizations in the group. The size of the link between two groups is proportional to the number of links from the organizations in one group to the organizations in the other group in the original network.
